# New insights into phylogeography of worldwide *Brucella canis* isolates by comparative genomics-based approaches: focus on Brazil

**DOI:** 10.1186/s12864-018-5001-6

**Published:** 2018-08-28

**Authors:** Acácia Ferreira Vicente, Guillaume Girault, Yannick Corde, Mateus Souza Ribeiro Mioni, Lara Borges Keid, Maryne Jay, Jane Megid, Virginie Mick

**Affiliations:** 1 EU/OIE/FAO & National Reference Laboratory for animal Brucellosis, Animal Health Laboratory, Paris-Est University/Anses, Maisons-Alfort, France; 2 Molecular Biology Laboratory, Dept. Veterinary Hygiene and Public Health, FMVZ, UNESP, Botucatu, Brazil; 3 Dept. Veterinary Medicine, University of Animal Science and Food Engineering, USP, Pirassununga, Brazil; 4 Present Address: Physiology of reproduction and behaviour joint research unit, INRA Val de Loire Centre, Nouzilly, France; 5 Present Address: Anses, Laboratoire de Lyon, UMR Mycoplasmoses des Ruminants, Lyon, France Université de Lyon, VetAgro Sup, UMR Mycoplasmoses des Ruminants, Marcy L’Etoile, France

**Keywords:** *Brucella canis*, Dog brucellosis, Brazil, São Paulo, Europe, Whole genome sequencing, SNP, Phylogeography

## Abstract

**Background:**

Canine brucellosis, due to *Brucella canis,* is a worldwide zoonosis that remains endemic in South America, including Brazil. Implementation of powerful whole-genome sequencing approaches allowed exploring the *Brucella* genus considered as monomorphic, with, to date, more than 500 genomes available in public databases*.* Nevertheless, with under-representation of *B. canis* genomes −only twenty complete or draft genomes−, lack of knowledge about this species is still considerable. This report describes a comparative genomics-based phylogeographic investigation of 53 *B. canis* strains, including 28 isolates paired-end sequenced in this work.

**Results:**

Obtained results allow identifying a SNP panel species-specific to *B. canis* of 1086 nucleotides. In addition, high-resolution analyses assess the epidemiological relationship between worldwide isolates. Our findings show worldwide strains are distributed among 2 distinct lineages. One of them seems to be specific to South American strains, including Brazil. *B. canis* South American strains may be identified by a SNP panel of 15 nucleotides, whereas a 22 SNP panel is sufficient to define contamination origin from Brazil. These results lead to the proposal of a possible spread route for dog brucellosis through South America. Additionally, whole-genome analyses highlight the remarkable genomic stability of *B. canis* strains over time and the sustainability of the infection in São Paulo over 12 year-period.

**Conclusions:**

Significant increase of *B. canis* genomes available in public databases provides new insights into *B. canis* infection in South America, including Brazil, as well as in the world, and also offers new perspectives for the *Brucella* genus *largo* sensu.

**Electronic supplementary material:**

The online version of this article (10.1186/s12864-018-5001-6) contains supplementary material, which is available to authorized users.

## Background

Canine brucellosis is a worldwide zoonosis caused by *Brucella canis* [1–3]. This bacterium is usually associated with dogs and occasionally causes brucellosis in humans [1]. It is considered as the main cause of reproductive failure in dogs, and responsible for important economic losses to kennels [3, 4].

*B. canis* infections, mainly diagnosed by serological methods and bacteriological evidence [5], remain endemic in South America, including Brazil with high dog population [3], raising public and animal health concerns. Although some studies have emphasized clinical and epidemiological importance of *B. canis* in the canine population, especially in confinement areas, such as kennels that increase the probability of disease transmission [6], canine brucellosis is underestimated in animals and human and its epidemiological aspects are still poorly understood.

*Brucella* is a well-known genus, whose taxonomy is in constant evolution, with the ongoing description of new species [7–9]. To date, *Brucella* genomes are composed of 2 circular chromosomes, except for *B. suis* biovar 3 (only 1 chromosome), with a global genome size of approximately 3.3 Mb [10]. All *Brucella* species are highly related to each other genetically, with sequence similarity values of 98% to 100% in the core genome [11]. Despite its genetic homogeneity, genotyping and phylogenetic approaches based on multiple genomic markers, like MLST −Multi Locus Sequence Typing−[12] or MLVA −Multiple-Locus Variable number tandem repeat Analysis−[13], are robust tools for wide- and fine-scale epidemiological/taxonomical investigations. Moreover, high-resolution studies based on *Brucella* whole genome strategies have been reported most recently, providing new insights into the genus [14–19]. Nevertheless, lack of knowledge about *B. canis* is still considerable. Indeed, although more than 500 *Brucella* genomes are available on public databases, *B. canis* is poorly represented with only 20 complete and draft genomes available [20–25].

The purpose of this work was a comparative genomics-based phylogeographic investigation of 27 worldwide *B. canis* field strains, with a focus on Brazil, in order to improve knowledge about *B. canis* epidemiology in the world, and especially in South America.

## Methods

### *Brucella* strains

Twenty-eight dog and human *B. canis* strains −including the *B. canis* reference strain Rm6–66 (ATCC 23365)−were isolated from routine epidemiological veterinary investigations conducted in São Paulo, Brazil, in 2005 and 2015 (Keid, L.B., personal data) or were obtained from the ANSES collection, especially from Europe (Additional file 1: Table S1).

### Phenotypic characterization

All isolates were confirmed as *B. canis* using conventional *Brucella* typing methods, based on CO_2_ requirement, H_2_S production, oxidase test, urea hydrolysis, agglutination with monospecific sera, fuchsin, thionin and safranin dye sensitivity and phage typing [26].

### Molecular studies

Genomic DNA was extracted from *Brucella* cultures using the High Pure PCR Template Preparation Kit (Roche Diagnostics, France), according to the manufacturer’s instructions. Molecular species confirmation was performed by Suis-Ladder multiplex PCR assay as previously described [27].

In this study, 28 *B. canis* strains (Additional file 1: Table S1), including the *B. canis* reference strain Rm6–66 (ATCC 23365), were paired-end sequenced at the Genoscreen institute (Lille, France) on Illumina platforms −HiSeq2000 and MiSeq (2 × 250 bp)−. An average 50-fold sequencing depth was obtained (100-fold coverage for Rm6–66). In addition, complete and draft genomes, as well as Sequence Read Archives (SRA, raw reads), belonging to the *B. canis* species (*n* = 25) were retrieved from Pathosystems Resource Integration Center (PATRIC) and NCBI center (Additional file 1: Table S1).

To discard misassemblies, raw sequencing reads were trimmed using Trimmomatic-0.36 (phred33, minimum 50 bp length). Chimeric genomes from databases have been generated by merging both chromosomes 1 and 2 to compare complete and draft genomes. To homogenize data, sets of sequencing pseudo-reads were created in silico on contigs from public databases using ART program with a 250 bp length and a 50-fold depth [28]. The mapping step of short read datasets on FASTQ format from this study as well as from pseudo-reads generated with ART was realized using BWA algorithm implemented in BioNumerics 7.6.1 (Applied Maths, Belgium) with 90% sequence similarity against the Rm6–66 *B. canis* reference genome (CP007758.1, CP007759.1). A set of SNPs was determined for each genome sequencing data respect to the Rm6–66 genome, using the BioNumerics whole genome SNP (wgSNP) module. Several position filters were applied on the SNP matrix: (*i*) contiguous SNPs were removed (if found in a 10 bp-window), (*ii*) position mask for repeated elements, including tandem repeats, (*iii*) a minimum 20-fold coverage for each SNP was required, and (*iv*) ambiguous (i.e. non-ACGT bases) and unreliable bases (i.e. Ns) were discarded. The refined SNP matrix was used to generate a minimum spanning tree using maximum bootstrap maximum likelihood approach, allowing phylogenetic analyses.

### Nucleotide sequence accession numbers

Raw sequencing data of five representative strains of this whole-genome sequencing project (PRJEB22763) have been deposited in the European Nucleotide Archive (ENA) (http://www.ebi.ac.uk/ena/data/view/PRJEB22763) under accession numbers ERR2136545, ERR2136546, ERR2136547, ERR2136548 and ERR2136549 (Additional file 1: Table S1).

## Results

**Fig. 1:**
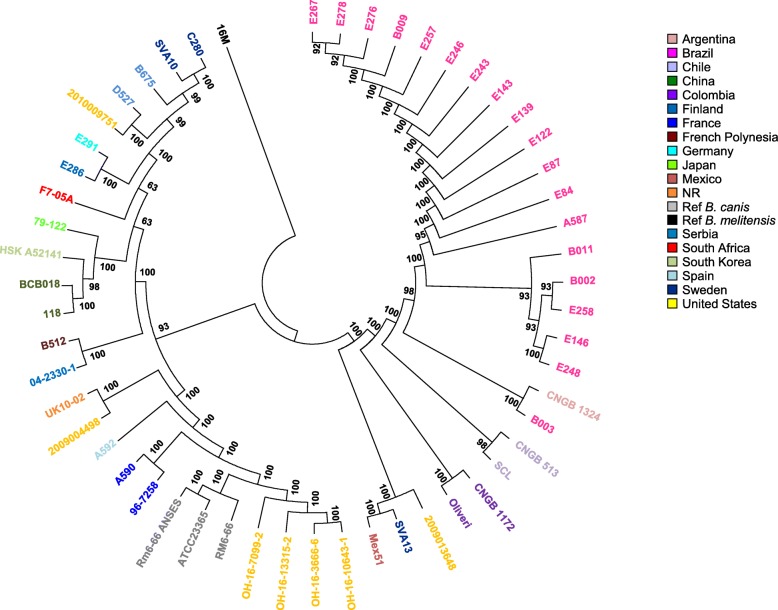
Comparative genomics-based analyses of 53 *B. canis* strains. This maximum likelihood tree was generated from 7327 SNPs identified from complete genomes of 53 *B. canis* strains and the reference strain *B. melitensis* biovar 1 16 M. Color codes represent geographic origins. The length of each branch is proportional (logarithmic scale) to the indicated number of SNPs. The tree size is 7345, indicating a very small part of homoplasia in the tree (0.25%)

All isolates investigated in this study were confirmed as *B. canis* by phenotypic and molecular (Suis-Ladder) approaches (data not shown). A total of 27 *B. canis* field strains, as well as the *B. canis* reference strain Rm6–66, were characterized by paired-end WGS. After applying different filters, a maximum likelihood tree (Fig. 1) was generated from 53 *B. canis* complete genomes and rooted with the reference strain *B. melitensis* biovar 1 16 M. A 0.25% homoplasia was considered. Bootstrap values supported strong confidence for each branch (mean: 97.43%; intervals 63–100%). A total of 7327 chromosomal SNPs were identified, including 1086 nucleotides specific to *B. canis*.

The *B. canis* investigated strains were distributed into two distinct clades (Fig. 1). The first clade, named here lineage 1 (*n* = 26), was mainly composed of strains isolated in Europe, Asia and USA, including the reference strain. Lineage 1 diverged from the most recent common ancestor −MRCA−of all *B. canis* isolates with only 3 SNPs. Two branches were then identified and diverged from the node with similar SNP numbers (17 and 21 SNPs). The second clade, named here lineage 2 (*n* = 27), contained all the strains isolated from South America (Argentina, Brazil, Chile, Colombia), as well as 2 North and Central American strains (USA, Mexico) and only one from Europe (Sweden).

### Asia

Four Asian strains were represented in this study, isolated from China (*n* = 2), Japan (*n* = 1) and South Korea (*n* = 1). They clustered together into a distinct sub-branch in lineage 1 (Fig. 1), among a polytomy of 3 branches, including too a sub-branch with European isolates (Finland, Sweden, Serbia, and Germany) and one American isolate (USA), and a sub-branch in singleton (South Africa). These four strains analyzed in this study seemed to be defined by a specific SNP panel of 12 markers.

### Europe

All strains isolated from Europe included in this study (*n* = 11), except for one, were clustered in lineage 1 (Fig. 1). Curiously, only one strain from Sweden −SVA13− was identified in lineage 2, 87 SNP distant from MRCA. This strain has been isolated from a dog imported from Spain for breeding [23]. Interestingly, one other Spanish strain −A592−was included in this comparative analysis. It clustered in lineage 1, together with French isolates, reference strains and USA strains, respectively with a distance of 69, 70 and 73 SNPs. Similarly, the *B. canis* German strain E291 was close to a Serbian strain −E286−with 23 SNP difference, whereas a 105 SNP difference was observed with another Serbian strain 04–2330-1 (strain from ANSES collection previously sequenced by Broad Institute Center). Finnish strains exhibited too some differences, with a 78 SNP distance from each other. These results might suggest the existence of regional polymorphism or the circulation of different *B. canis* clones among a same country.

### North America

Seven strains from North America (USA) were included in this study. Surprisingly, the great majority (*n* = 6) clustered into lineage 1 (Fig. 1), and only one strain −2,009,013,648−[25] isolated from Arizona in 2009 belonged to lineage 2, including all South and Central American strains (Fig. 1). Interestingly, Arizona shares common boundaries with Mexico. Regarding closeness of both strains, results suggest a same cross-border contamination origin.

Within lineage 1, the distribution of USA strains was heterogeneous since strains belonged to distinct sub-clusters −e.g. strains 2,010,009,751 and 2,009,004,498 respectively isolated from Massachusetts in 2010 and from Louisiana in 2009 [25] were 140 SNP distant between each other−. Although almost 40 *B. canis* raw sequencing data have recently been added to NCBI SRA (PRJNA369091), faced with lack of information and metadata poorly filled, only 4 of these strains have been included in this study. They showed weak polymorphism (distance between furthest strains = 32 SNPs) and clustered together with *B. canis* reference genomes.

Despite possible sampling bias, WgSNP results seemed to indicate an important genetic diversity of *B. canis* strains circulating in USA.

### Central and South America

This study describes a comparative genomics-based investigation of Brazilian *B. canis* strains. To date, lack of knowledge regarding *B. canis* epidemiology in South America, and especially in Brazil, is reported.

**Fig. 2:**
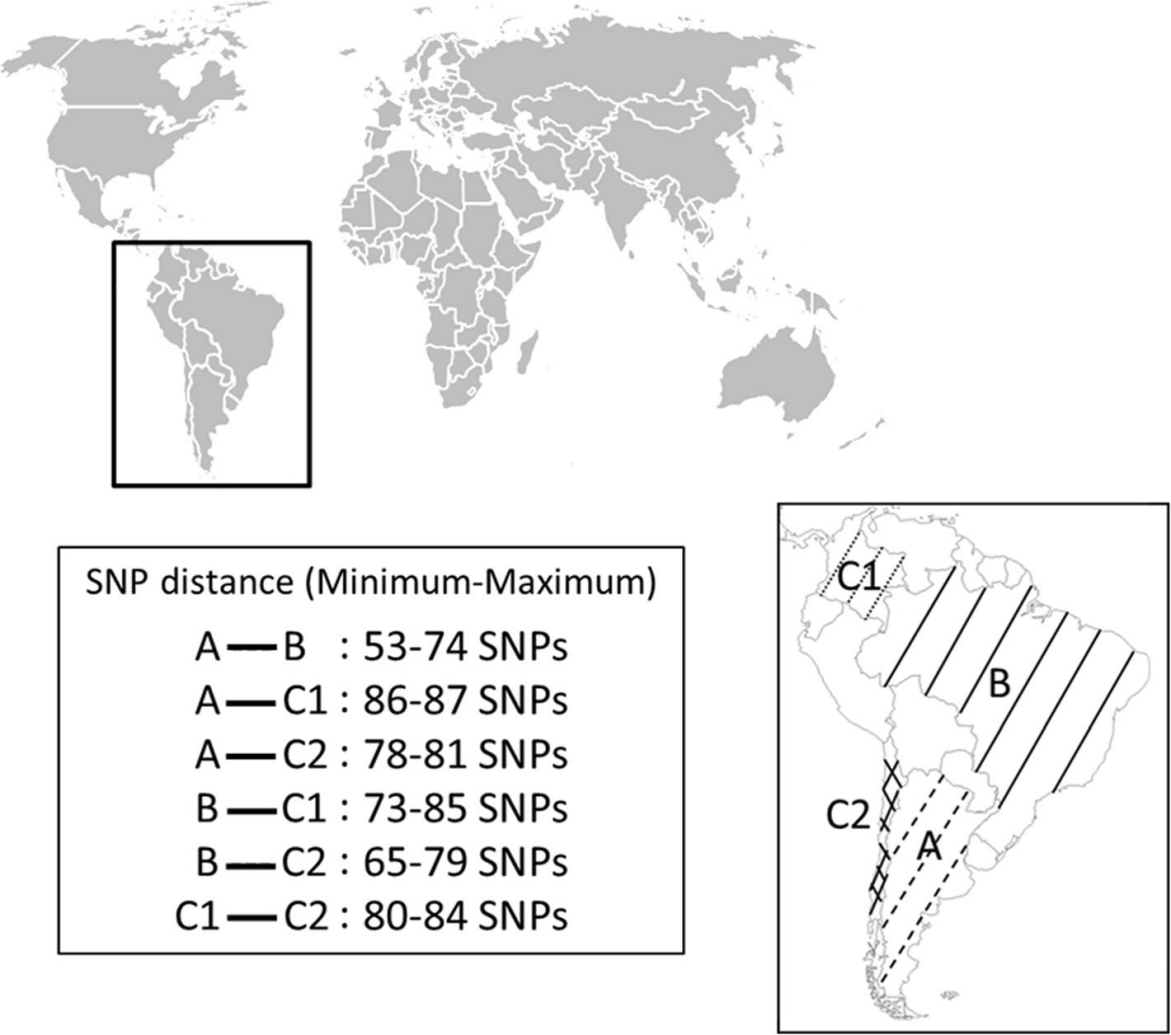
SNP distance between different *B. canis* strains isolated from South America. This figure indicates the distance observed in SNPs between the strains isolated from South America in 4 different countries. A: Argentina (dashed lines); B: Brazil (full lines); C1: Colombia (dotted lines): C2: Chile (crossed lines). [Blank map available on Wikimedia commons]

All *B. canis* strains isolated from South and Central America were located among lineage 2 (Fig. 1). Lineage 2 split into two distinct branches. The minor one contained the Mexican isolate Mex51, clustered together with an USA isolate and a Swedish strain imported from Spain. The major branch allowed grouping all South American strains into a unique, exclusive and homogeneous cluster, with a node that could represent the MRCA of South American *B. canis* strains −SA-MRCA−. The two most distant strains among South America isolates harbored a 87 SNP difference (Fig. 2).

This South American branch split in two sub-branches. A first sub-branch contained strains isolated from Colombia −Oliveri and CNGB 1172−and radiated respectively 37 and 38 SNPs away from SA-MRCA. Both Colombian isolates were very similar, with only 3 SNP difference despite isolation from different hosts (human and dog, respectively for CNGB 1172 and for Oliveri), suggesting contamination by the same strain. A second sub-branch radiated 7 SNPs away from SA-MRCA and split in 3 sub-clusters. Except for two singletons, isolated from Chile −SCL and CNGB 513−, only Brazilian strains, as well as one Argentinian strain (CNGB 1324) constituted this sub-branch. Interestingly, the strain B003 isolated from South Brazil, adjacent to Argentina, seemed closer to the Argentina strain than other Brazilian strains, with a minimal distance of 50 SNPs, suggesting an infection contamination from Argentina to Brazil. All other Brazilian strains formed a single complex, split into 2 branches that have diverged very early (1 and 2 SNPs) and harbored very weak polymorphism (maximal distance of 25 SNPs). In addition, length of different branches identified in this study varied very little, from 3 to 14 SNPs, even over a 17 year-period (1998–2015).

Thus, a 12 SNP panel seemed to be sufficient to characterize the *B. canis* South American strains, although *B. canis* contamination origin from Brazil might be identified using a 22 SNP panel (Additional file 2: Table S2).

### Focus on São Paulo (Brazil)

Seventeen *B. canis* isolates collected from São Paulo, Brazil, over 12-year-period (2003–2015) were investigated in this study. Fourteen were isolated from three well-identified kennels, respectively both in 2005 (k1, k2) and one in 2015 (k3), and three strains were isolated in 2003 from not reported kennels (Additional file 1: Table S1). Interestingly, no trade/historical exchange between kennels was reported.

As expected, wgSNP analyses showed a minor SNP difference, independent of isolation year, between São Paulo strains, ranging from 3 to 25 nucleotides (Fig. 1). Most *B. canis* strains isolated among a same kennel were strictly identical or harbored no significant difference, as strains collected in the same isolation year (e.g. E087, E122 from k1 and E143, E243, E246, E257 from k2).

## Discussion

To improve understanding of the genetic diversity of *B. canis*, we genotyped 27 worldwide *B. canis* field isolates by performing whole genome SNP analysis. Indeed, this approach is able to infer the relationships among worldwide *B. canis* isolates.

Comparative genomic analysis of 53 dog and human *B. canis* strains was performed. Robustness of the wgSNP method applied in this study was assessed by sequencing data comparison of 2 isolates from ANSES collection (Additional file 1: Table S1), independently sequenced in this study and previously by the Broad Institute center. Thus, C280 and SVA10 strains showed a strictly identical genome, as well as A590 and 96–7258 genomes harbored only one SNP difference. These results highlighted in vitro genomic stability of *B. canis* genomes despite successive cultural steps susceptible to induce several mutational events. Similarly and unsurprisingly, wgSNP results showed too extreme in vivo genomic stability over time, e.g. two samples isolated in China (BCB018 and 118) [20, 21] with only 12 SNP difference in a 20 year-interval.

No host specificity was observed. Nevertheless, SNP analysis indicated a spatial distribution of the isolates that could not be correlated with a time-span scale. A slight, almost inexistent genetic diversity was observed among Brazilian isolates. According to the more parsimonious hypothesis, our results suggested one single introduction could have led to the divergence of two Brazilian sub-branches observed here. Moreover, the short branch length, even over a long time-period, suggested circulation of a dominant clone in South America and emphasizing genetic stability of *B. canis* genomes over time. It is interesting to note *B. canis* South American strains could be identified by a SNP panel of 12 nucleotides, whereas a 22 SNP panel is sufficient to refine Brazilian contamination origin of *B. canis* strains, assuming contamination origin of Brazilian B003 strain is from Argentina.

Here, we investigated potential genetic diversity over 12-year-period in a restricted geographical area: from different São Paulo kennels. Absence of significant difference whatever the isolation year between *B. canis* studied isolates suggested a same contamination origin and/or the circulation of a dominant clone. In addition, the distance observed over a 10-year period between strains isolated from a same location was not greater (e.g. E267 vs E258, or E267 vs B009), suggesting the sustainability of *B. canis* infection over time in São Paulo.

The potential of whole-genome sequencing leads to propose a possible spread route for dog brucellosis through South America. Indeed, our findings suggested the presence of *B. canis* in South America probably might be resulted from an introduction from USA and/or from Mexico (Fig. 1) to Colombia (maximum distance of 84, 85 and 87 SNPs respectively with Chile, Brazil and Argentina), followed by spread to Brazil, Argentina and Chile (Fig. 2). On the current state of available genome dataset −Argentina, Chile and Brazil are part of a polytomic branch (Fig. 1)−, it is difficult to hypothesize into the details this second evolving step of the *B. canis* infection through South America. Indeed, a largest dataset of representative strains of these countries is required to propose a strongest hypothesis.

## Conclusions

This report describes a comparative genomics-based phylogeographic investigation of *B. canis* field strains isolated from Brazil. Results obtained allow assessing the epidemiological relationship between worldwide strains and hypothesizing a possible spread route for dog brucellosis through South America. In addition, whole genome analyses highlight the remarkable genomic stability of *B. canis* strains over time and the sustainability of the infection in São Paulo.

Significant increase of *B. canis* genomes available in public databases, resulting from this work, provides new insights not only into *B. canis* infection in South America, including Brazil, as well in the world, but also offers new perspectives for the *Brucella* genus *largo* sensu.

## Additional files

Additional file 1: **Table S1.** *B. canis* strains investigated in this study and from public databases and whole sequencing data. (DOCX 19 kb)

Additional file 2 **Table S2.** Determination of 22 SNP panel specific to Brazilian *B. canis* strains. (DOCX 37 kb)

## Abbreviations

MLST: Multi Locus Sequence Typing
MLVA: Multiple-Locus Variable number tandem repeat Analysis
MRCA: Most Recent Common Ancestor
SNP: Single Nucleotide Polymorphism
WGS: Whole Genome Sequencing
wgSNP: Whole genome SNP
