# Supplementary material for: New insights into phylogeography of worldwide Brucella canis isolates by comparative genomics-based approaches: focus on Brazil
Source: BMC Genomics. 2018 Aug 28;19:636. doi: 10.1186/s12864-018-5001-6 (PMC6114238; doi:10.1186/s12864-018-5001-6)
Supplement: Supplementary file 1 — Table S1. B. canis strains investigated in this study and from public databases and whole sequencing data. (DOCX 19 kb) [file 12864_2018_5001_MOESM1_ESM.docx]

Supplementary Table 1: *B. canis* strains investigated in this study and from public databases and whole sequencing data

| **Key** | **Id Strain** | **Host** | **Year** | **Country** | **Region** | **Sequencing Depth (x-fold)** | **Accession number** | **Reference** |
| --- | --- | --- | --- | --- | --- | --- | --- | --- |
| Rm6-66 |  | NR | NR | NR | NR | 100 |  | this study |
| A587 | 03-2770-12 | Dog | 2003 | Brazil | NR | 54 |  | this study |
| A590 | 96-7258 | Dog | 1996 | France | NR | 57 |  | this study |
| A592 | 96-9626 | Dog | 1996 | Spain | NR | 47 | ERR2136545 | this study |
| B002 | 07-2859-6070 | Dog | 1998 | Brazil | NR | 46 | ERR2136546 | this study |
| B003 | 07-2859-6071 | Dog | 1995 | Brazil | NR | 39 | ERR2136547 | this study |
| B009 | 07-2859-6077 | Dog | 2003 | Brazil | São Paulo | 26 |  | this study |
| B011 | 07-2859-6083 | Dog | 2003 | Brazil | São Paulo | 28 |  | this study |
| B512 | 08-1276-2270 | Human | 2008 | French Polynesia | NR | 36 |  | this study |
| B675 | 09-369-776(1) | Dog | 2009 | Finland | NR | 38 | ERR2136548 | this study |
| C280 | 11-1961-3694(1) | Dog | 2011 | Sweden | NR | 35 |  | this study |
| D527 | 14-901-3115 | Dog | 2014 | Finland | NR | 44 |  | this study |
| E084 | 10466 | Dog | 2005 | Brazil | São Paulo-k1 | 92 |  | this study |
| E087 | 10469 | Dog | 2005 | Brazil | São Paulo-k1 | 34 | ERR2136549 | this study |
| E122 | 10473 | Dog | 2005 | Brazil | São Paulo-k1 | 39 |  | this study |
| E139 | 10486 | Dog | 2005 | Brazil | São Paulo-k1 | 47 |  | this study |
| E143 | 10490 | Dog | 2005 | Brazil | São Paulo-k2 | 46 |  | this study |
| E146 | 10493 | Dog | 2005 | Brazil | São Paulo-k2 | 27 |  | this study |
| E243 | 10499 | Dog | 2005 | Brazil | São Paulo-k2 | 42 |  | this study |
| E246 | 10502 | Dog | 2005 | Brazil | São Paulo-k2 | 31 |  | this study |
| E248 | 10504 | Dog | 2005 | Brazil | São Paulo-k2 | 39 |  | this study |
| E257 | 10509 | Dog | 2005 | Brazil | São Paulo-k2 | 39 |  | this study |
| E258 | 10510 | Dog | 2005 | Brazil | São Paulo-k2 | 32 |  | this study |
| E267 | 10519 | Dog | 2015 | Brazil | São Paulo-k3 | 33 |  | this study |
| E276 | 10522 | Dog | 2015 | Brazil | São Paulo-k3 | 33 |  | this study |
| E278 | 10524 | Dog | 2015 | Brazil | São Paulo-k3 | 39 |  | this study |
| E286 | 04-2330-4 | Dog | 2004 | Serbia | NR | 34 |  | this study |

| E291 | 00-5732-9911 | Dog | 2000 | Germany | NR | 28 |  | this study |
| --- | --- | --- | --- | --- | --- | --- | --- | --- |
| 04-2330-1 | 04-2330-1 | Dog | NR | Serbia | NR | 79 | AXNG00000000 |  |
| 118 | 118 | Human | 2008 | China | NR | 100 | AMOZ00000000 | (20) |
| 79/122 | 79/122 | Dog | 1979 | Japan | NR | 168 | AQJY00000000 |  |
| 96-7258 | 96-7258 | Dog | NR | France | NR | 79 | AXNF00000000 |  |
| ATCC23365 | ATCC23365 | NR | NR | NR | NR | NR | CP000872, CP000873 |  |
| BCB018 | BCB018 | Dog | 1988 | China | NR | 180 | ALOJ00000000 | (21) |
| CNGB 1172 | CNGB 1172 | Human | 2006 | Colombia | NR | 169 | AQMY00000000 |  |
| CNGB 1324 | CNGB 1324 | Human | 2008 | Argentina | NR | 171 | AQMZ00000000 |  |
| CNGB 513 | CNGB 513 | Human | 2001 | Chile |  | 168 | AQJZ00000000 |  |
| F7/05A | F7/05A | Dog | 2005 | South Africa | NR | 172 | AQNA00000000 |  |
| HSK A52141 | HSK A52141 | Dog | NR | South Korea | Hwaseong | NR | CP003174, CP003175 | (22) |
| Oliveri | Oliveri | Dog | NR | Colombia | NR | 28 | HG803176.1, HG803175.1 | (17) |
| SCL | SCL | NR | 2008 | Chile | NR | 145 | LGAQ01000000 |  |
| RM6-66 | RM6-66 | NR | NR | NR | NR | 320 | CP007758.1, CP007759.1 | (24) |
| SVA13 | SVA13 | Dog | 2013 | Sweden | NR | 62 | CP007630.1, CP007629.1 | (23) |
| UK10/02 | UK10/02 | NR | 2002 | NR | NR | 171 | AQNB00000000 |  |
| SVA10 | SVA10 | Dog | 2010 | Sweden | NR | 70 | MAXW00000000 |  |
| 2010009751 | 2010009751 | Dog | 2010 | United States | Massachusetts | 49 | CP016977, CP016978 | (25) |
| 2009013648 | 2009013648 | Human | 2009 | United States | Arizona | 49 | CP016975, CP016976 | (25) |
| 2009004498 | 2009004498 | Human | 2009 | United States | Louisiana | 21 | CP016973, CP016974 | (25) |
| OH-16-10643-1 | OH-16-10643-1 | NR | 2016 | United States |  |  | SRR5484407 |  |
| OH-16-3666-6 | OH-16-3666-6 | NR | 2016 | United States |  |  | SRR5483857 |  |
| OH-16-13315-2 | OH-16-13315-2 | NR | 2016 | United States |  |  | SRR5484408 |  |
| OH-16-7099-2 | OH-16-7099-2 | NR | 2016 | United States |  |  | SRR5484406 |  |
| Mex51 | Mex51 | NR | NR | Mexico |  |  | SRR4039007 |  |

Table footnotes: NR: Not Reported; k: kennel.
