# Supplementary material for: New insights into phylogeography of worldwide Brucella canis isolates by comparative genomics-based approaches: focus on Brazil
Source: BMC Genomics. 2018 Aug 28;19:636. doi: 10.1186/s12864-018-5001-6 (PMC6114238; doi:10.1186/s12864-018-5001-6)
Supplement: Supplementary file 2 — Table S2. Determination of 22 SNP panel specific to Brazilian B. canis strains. (DOCX 37 kb) [file 12864_2018_5001_MOESM2_ESM.docx]

**Supplementary Table 2:**  Determination of 22 SNP panel specific to Brazilian *B. canis* strains.

| **Genome position** | **53452** | **109152** | **258173** | **304079** | **336414** | **379181** | **883340** | **920253** | **1393000** | **1427203** | **1475715** | **1634459** | **1926525** | **2116714** | **2172616** | **2303720** | **2315427** | **2506475** | **2889459** | **2915327** | **3193383** | **3261788** |
| --- | --- | --- | --- | --- | --- | --- | --- | --- | --- | --- | --- | --- | --- | --- | --- | --- | --- | --- | --- | --- | --- | --- |
| 16M | C | T | g | C | g | g | A | C | g | g | C | C | g | g | g | g | C | g | g | C | g | C |
| ATCC 23365 | C | T | g | C | g | g | A | C | g | g | C | C | g | g | g | g | C | g | g | C | g | C |
| RM6/66 | C | T | g | C | g | g | A | C | g | g | C | C | g | g | g | g | C | g | g | C | g | C |
| **Rm6-66/ANSES** | C | T | g | C | g | g | A | C | g | g | C | C | g | g | g | g | C | g | g | C | g | C |
| 04-2330-1 | C | T | g | C | g | g | A | C | g | g | C | C | g | g | g | g | C | g | g | C | g | C |
| 79/122 | C | T | g | C | g | g | A | C | g | g | C | C | g | g | g | g | C | g | g | C | g | C |
| 96-7258 | C | T | g | C | g | g | A | C | g | g | C | C | g | g | g | g | C | g | g | C | g | C |
| 118 | C | T | g | C | g | g | A | C | g | g | C | C | g | g | g | g | C | g | g | C | g | C |
| 2009004498 | C | T | g | C | g | g | A | C | g | g | C | C | g | g | g | g | C | g | g | C | g | C |
| 2009013648 | C | T | g | C | g | g | A | C | g | g | C | C | g | g | g | g | C | g | g | C | g | C |
| 2010009751 | C | T | g | C | g | g | A | C | g | g | C | C | g | g | g | g | C | g | g | C | g | C |
| BCB018 | C | T | g | C | g | g | A | C | g | g | C | C | g | g | g | g | C | g | g | C | g | C |
| CNGB 513 | C | T | g | C | g | g | A | C | g | g | C | C | g | g | g | g | C | g | g | C | g | C |
| CNGB 1172 | C | T | g | C | g | g | A | C | g | g | C | C | g | g | g | g | C | g | g | C | g | C |
| CNGB 1324 | C | T | g | C | g | g | A | C | g | g | C | C | g | g | g | g | C | g | g | C | g | C |
| F7/05A | C | T | g | C | g | g | A | C | g | g | C | C | g | g | g | g | C | g | g | C | g | C |
| HSK A52141 | C | T | g | C | g | g | A | C | g | g | C | C | g | g | g | g | C | g | g | C | g | C |
| Oliveri | C | T | g | C | g | g | A | C | g | g | C | C | g | g | g | g | C | g | g | C | g | C |
| SCL | C | T | g | C | g | g | A | C | g | g | C | C | g | g | g | g | C | g | g | C | g | C |
| SVA13 | C | T | g | C | g | g | A | C | g | g | C | C | g | g | g | g | C | g | g | C | g | C |
| SVA10 | C | T | g | C | g | g | A | C | g | g | C | C | g | g | g | g | C | g | g | C | g | C |
| UK10/02 | C | T | g | C | g | g | A | C | g | g | C | C | g | g | g | g | C | g | g | C | g | C |
| OH-16-7099-2 | C | T | g | C | g | g | A | C | g | g | C | C | g | g | g | g | C | g | g | C | g | C |
| OH-16-10643-1 | C | T | g | C | g | g | A | C | g | g | C | C | g | g | g | g | C | g | g | C | g | C |
| OH-16-13315-2 | C | T | g | C | g | g | A | C | g | g | C | C | g | g | g | g | C | g | g | C | g | C |
| OH-16-3666-6 | C | T | g | C | g | g | A | C | g | g | C | C | g | g | g | g | C | g | g | C | g | C |
| Mex51 | C | T | g | C | g | g | A | C | g | g | C | C | g | g | g | g | C | g | g | C | g | C |
| **A587** | **T** | **C** | **C** | **g** | **A** | **T** | **g** | **T** | **A** | **A** | **T** | **T** | **A** | **A** | **A** | **A** | **T** | **A** | **A** | **T** | **T** | **T** |
| **E139** | **T** | **C** | **C** | **g** | **A** | **T** | **g** | **T** | **A** | **A** | **T** | **T** | **A** | **A** | **A** | **A** | **T** | **A** | **A** | **T** | **T** | **T** |
| **B009** | **T** | **C** | **C** | **g** | **A** | **T** | **g** | **T** | **A** | **A** | **T** | **T** | **A** | **A** | **A** | **A** | **T** | **A** | **A** | **T** | **T** | **T** |
| **E267** | **T** | **C** | **C** | **g** | **A** | **T** | **g** | **T** | **A** | **A** | **T** | **T** | **A** | **A** | **A** | **A** | **T** | **A** | **A** | **T** | **T** | **T** |
| **E276** | **T** | **C** | **C** | **g** | **A** | **T** | **g** | **T** | **A** | **A** | **T** | **T** | **A** | **A** | **A** | **A** | **T** | **A** | **A** | **T** | **T** | **T** |
| **E278** | **T** | **C** | **C** | **g** | **A** | **T** | **g** | **T** | **A** | **A** | **T** | **T** | **A** | **A** | **A** | **A** | **T** | **A** | **A** | **T** | **T** | **T** |
| **E084** | **T** | **C** | **C** | **g** | **A** | **T** | **g** | **T** | **A** | **A** | **T** | **T** | **A** | **A** | **A** | **A** | **T** | **A** | **A** | **T** | **T** | **T** |
| **E087** | **T** | **C** | **C** | **g** | **A** | **T** | **g** | **T** | **A** | **A** | **T** | **T** | **A** | **A** | **A** | **A** | **T** | **A** | **A** | **T** | **T** | **T** |
| **E122** | **T** | **C** | **C** | **g** | **A** | **T** | **g** | **T** | **A** | **A** | **T** | **T** | **A** | **A** | **A** | **A** | **T** | **A** | **A** | **T** | **T** | **T** |
| **E143** | **T** | **C** | **C** | **g** | **A** | **T** | **g** | **T** | **A** | **A** | **T** | **T** | **A** | **A** | **A** | **A** | **T** | **A** | **A** | **T** | **T** | **T** |
| **E243** | **T** | **C** | **C** | **g** | **A** | **T** | **g** | **T** | **A** | **A** | **T** | **T** | **A** | **A** | **A** | **A** | **T** | **A** | **A** | **T** | **T** | **T** |
| **E246** | **T** | **C** | **C** | **g** | **A** | **T** | **g** | **T** | **A** | **A** | **T** | **T** | **A** | **A** | **A** | **A** | **T** | **A** | **A** | **T** | **T** | **T** |
| **E257** | **T** | **C** | **C** | **g** | **A** | **T** | **g** | **T** | **A** | **A** | **T** | **T** | **A** | **A** | **A** | **A** | **T** | **A** | **A** | **T** | **T** | **T** |
| **E258** | **T** | **C** | **C** | **g** | **A** | **T** | **g** | **T** | **A** | **A** | **T** | **T** | **A** | **A** | **A** | **A** | **T** | **A** | **A** | **T** | **T** | **T** |
| **B002** | **T** | **C** | **C** | **g** | **A** | **T** | **g** | **T** | **A** | **A** | **T** | **T** | **A** | **A** | **A** | **A** | **T** | **A** | **A** | **T** | **T** | **T** |
| **B011** | **T** | **C** | **C** | **g** | **A** | **T** | **g** | **T** | **A** | **A** | **T** | **T** | **A** | **A** | **A** | **A** | **T** | **A** | **A** | **T** | **T** | **T** |
| **E146** | **T** | **C** | **C** | **g** | **A** | **T** | **g** | **T** | **A** | **A** | **T** | **T** | **A** | **A** | **A** | **A** | **T** | **A** | **A** | **T** | **T** | **T** |
| **E248** | **T** | **C** | **C** | **g** | **A** | **T** | **g** | **T** | **A** | **A** | **T** | **T** | **A** | **A** | **A** | **A** | **T** | **A** | **A** | **T** | **T** | **T** |
| **A590** | C | T | g | C | g | g | A | C | g | g | C | C | g | g | g | g | C | g | g | C | g | C |
| **A592** | C | T | g | C | g | g | A | C | g | g | C | C | g | g | g | g | C | g | g | C | g | C |
| **B003** | **C** | **T** | **g** | **C** | **g** | **g** | **A** | **C** | **g** | **g** | **C** | **C** | **g** | **g** | **g** | **g** | **C** | **g** | **g** | **C** | **g** | **C** |
| **B512** | C | T | g | C | g | g | A | C | g | g | C | C | G | g | g | g | C | g | g | C | g | C |
| **B675** | C | T | g | C | g | g | A | C | g | g | C | C | G | g | g | g | C | g | g | C | g | C |
| **C280** | C | T | g | C | g | g | A | C | g | g | C | C | G | g | g | g | C | g | g | C | g | C |
| **D527** | C | T | g | C | g | g | A | C | g | g | C | C | G | g | g | g | C | g | g | C | g | C |
| **E286** | C | T | g | C | g | g | A | C | g | g | C | C | G | g | g | g | C | g | g | C | g | C |
| **E291** | C | T | g | C | g | g | A | C | g | g | C | C | G | g | g | g | C | g | g | C | g | C |

Table footnotes: Strains indicated in bold were sequenced in this study. Brazilian *B. canis* isolates are represented by grey color. Genome positions match with *B. melitensis* biovar 1 16 M genome.
